# Dice-XMBD: Deep Learning-Based Cell Segmentation for Imaging Mass Cytometry

**DOI:** 10.3389/fgene.2021.721229

**Published:** 2021-09-15

**Authors:** Xu Xiao, Ying Qiao, Yudi Jiao, Na Fu, Wenxian Yang, Liansheng Wang, Rongshan Yu, Jiahuai Han

**Affiliations:** ^1^Department of Computer Science, School of Informatics, Xiamen University, Xiamen, China; ^2^National Institute for Data Science in Health and Medicine, Xiamen University, Xiamen, China; ^3^Aginome Scientific, Xiamen, China; ^4^School of Medicine, Xiamen University, Xiamen, China

**Keywords:** imaging mass cytometry, multiplexed imaging, single cell segmentation, U-net, knowledge distillation, digital pathology

## Abstract

Highly multiplexed imaging technology is a powerful tool to facilitate understanding the composition and interactions of cells in tumor microenvironments at subcellular resolution, which is crucial for both basic research and clinical applications. Imaging mass cytometry (IMC), a multiplex imaging method recently introduced, can measure up to 100 markers simultaneously in one tissue section by using a high-resolution laser with a mass cytometer. However, due to its high resolution and large number of channels, how to process and interpret the image data from IMC remains a key challenge to its further applications. Accurate and reliable single cell segmentation is the first and a critical step to process IMC image data. Unfortunately, existing segmentation pipelines either produce inaccurate cell segmentation results or require manual annotation, which is very time consuming. Here, we developed Dice-XMBD^1^, a Deep learnIng-based Cell sEgmentation algorithm for tissue multiplexed imaging data. In comparison with other state-of-the-art cell segmentation methods currently used for IMC images, Dice-XMBD generates more accurate single cell masks efficiently on IMC images produced with different nuclear, membrane, and cytoplasm markers. All codes and datasets are available at https://github.com/xmuyulab/Dice-XMBD.

## 1. Introduction

Analysis of the heterogeneity of cells is critical to discover the complexity and factuality of life system. Recently, single-cell sequencing technologies have been increasingly used in the research of developmental physiology and disease (Stubbington et al., 2017; Papalexi and Satija, 2018; Potter, 2018; Lähnemann et al., 2020), but the spatial context of individual cells in the tissue is lost due to tissue dissociation in these technologies. On the other hand, traditional immunohistochemistry (IHC) and immunofluorescence (IF) preserve spatial context but the number of biomarkers is limited. The development of multiplex IHC/IF (mIHC/mIF) technologies has enabled the simultaneous detection of multiple biomarkers and preserves spatial information, such as cyclic IHC/IF and metal-based multiplex imaging technologies (Zrazhevskiy and Gao, 2013; Angelo et al., 2014; Giesen et al., 2014; Tan et al., 2020). Imaging mass cytometry (IMC) (Giesen et al., 2014; Chang et al., 2017), one of metal-based mIHC technologies, uses a high-resolution laser with a mass cytometer and makes the measurement of 100 markers possible.

IMC has been utilized in studies of cancer and autoimmune disorders (Giesen et al., 2014; Damond et al., 2019; Ramaglia et al., 2019; Wang et al., 2019; Böttcher et al., 2020). Due to its high resolution and large number of concurrent marker channels available, IMC has been proven to be highly effective in identifying the complex cell phenotypes and interactions coupled with spatial locations. Thus, it has become a powerful tool to study tumor microenvironments and discover the underlying disease-relevant mechanisms (Brähler et al., 2018; Ali et al., 2020; Aoki et al., 2020; de Vries et al., 2020; Dey et al., 2020; Jackson et al., 2020; Zhang et al., 2020; Schwabenland et al., 2021). Apart from using IMC techniques alone, several other technologies, such as RNA detection *in situ* and 3D imaging, have been combined with IMC to expand its applicability and utility (Schulz et al., 2018; Bouzekri et al., 2019; Catena et al., 2020; Flint et al., 2020).

The IMC data analysis pipeline typically starts with single cell segmentation followed by tissue/cell type identification (Carpenter et al., 2006; Sommer et al., 2011; Liu et al., 2019). As the first step of an IMC data processing pipeline, the accuracy of single cell segmentation plays a significant role in determining the quality and the reliability of the biological results from an IMC study. Existing IMC cell segmentation methods include both unsupervised and supervised algorithms. Unsupervised cell segmentation, such as the watershed algorithm implemented in CellProfiler (Carpenter et al., 2006), does not require user inputs for model training. However, the segmentation results are not precise in particular when cells are packed closely or they are in complicated shapes. To achieve better segmentation results, supervised methods use a set of images annotated with pixel-level cell masks to train a segmentation classifier. However, the manual annotation task is very time consuming and expensive as well since it is normally done by pathologists or experienced staff with necessary knowledge in cell annotation. Particularly, for multiplexing cellular imaging methods such as IMC, their channel configurations including the total number of markers and markers selection are typically study dependent. Therefore, manual annotation may need to be performed repeatedly for each study to adapt the segmentation model to different IMC channel configurations, which can be impractical.

To overcome this limitation, a hybrid workflow combining unsupervised and supervised learning methods for cell segmentation was proposed (Ali et al., 2020). This hybrid workflow uses Ilastik (Sommer et al., 2011), an interactive image processing tool, to generate a probability map based on multiple rounds of user inputs and adjustments. In each round, a user only needs to perform a limited number of annotations on regions where the probability map generated based on previous annotations is not satisfactory. CellProfiler is then used to perform the single cell segmentation based on the probability map once the result from Ilastik is acceptable. This hybrid workflow significantly reduces manual annotation workload and has gained popularity in many recent IMC studies (Damond et al., 2019; Böttcher et al., 2020; de Vries et al., 2020; Jackson et al., 2020; Schwabenland et al., 2021). However, the annotation process still needs to be performed by experienced staff repeatedly for each IMC study, which is very inconvenient. In addition, the reproducibility of the experimental results obtained from this approach can be an issue due to the per-study, interactive training process used in creating the single cell masks. Hence, a more efficient, fully automated single cell segmentation method for IMC data without compromising the segmentation accuracy is necessary for IMC to gain broader applications in biomedical studies.

Convolutional neural networks (CNNs) have been successfully used for natural image segmentation and recently applied in biomedical image applications (Shen et al., 2017; Zhang et al., 2018; Andrade et al., 2019; Vicar et al., 2019). CNN-based U-Net was developed for pixel-wise cell segmentation of mammalian cells (Ronneberger et al., 2015). It has been demonstrated that the U-Net architecture and its variants such as Unet++ (Zhou et al., 2018), 3D Unet (Çiçek et al., 2016), and V-Net (Milletari et al., 2016) can obtain high segmentation accuracy. Motivated by the good performance of U-Nets in cell segmentation (Van Valen et al., 2016; Hollandi et al., 2020; Salem et al., 2020), we developed Dice-XMBD, a deep neural network (DNN)-based cell segmentation method for multichannel IMC images. Dice-XMBD is marker agnostic and can perform cell segmentation for IMC images of different channel configurations without modification. To achieve this goal, Dice-XMBD first merges multiple-channel IMC images into two channels, namely, a nuclear channel containing proteins originated from cell nucleus, and a cell channel containing proteins originated from cytoplasm and cell membrane. Channels of proteins with ambiguous locations are ignored by Dice-XMBD for segmentation as they contribute little to the segmentation results. Furthermore, to mitigate the annotation workload, we adopted the knowledge distillation learning framework (Hinton et al., 2015) in training Dice-XMBD, where the training labels were generated using Ilastik with interactive manual annotations as a teacher model. We used four IMC datasets of different channel configurations to evaluate the performance of Dice-XMBD and the results show that it can generate highly accurate cell segmentation results that are comparable to those from manual annotation for IMC images from both the same and different datasets to the training dataset, validating its applicability for generic IMC image segmentation tasks.

## 2. Materials and Methods

### 2.1. Overview of the Pipeline

**Figure 1:**
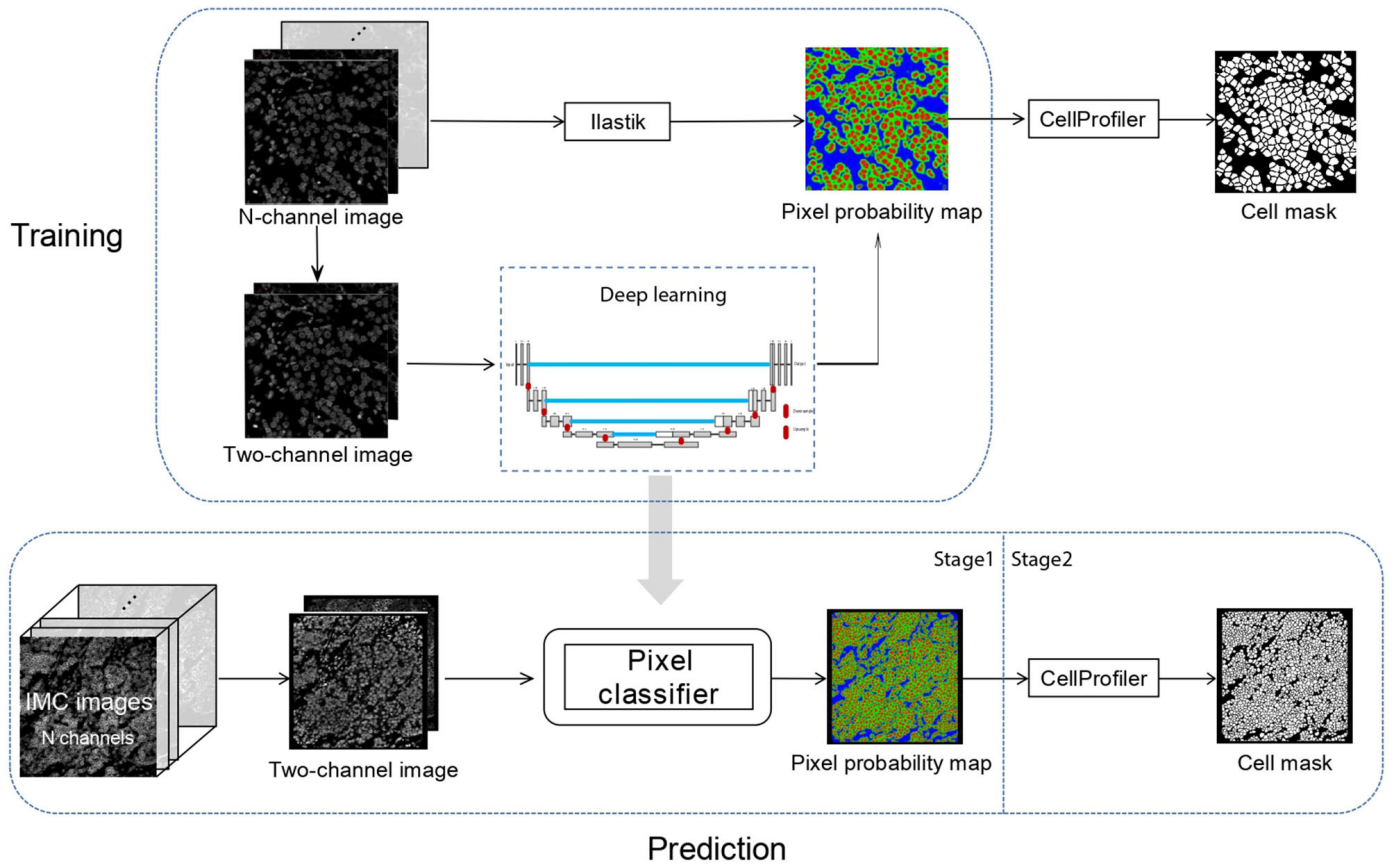
Dice-XMBD workflow. Imaging mass cytometry (IMC) images are combined into 2-channel images containing nuclear and membrane/cytoplasm proteins expression information. In stage 1, the pixel probability maps of the input 2-channel images are predicted using a semi-supervised learning model based on U-Net architecture. The training data were generated from Ilastik by an iterative interactive annotation process. In stage 2, the cell segmentation masks are generated from the pixel probability maps using the propagation method in CellProfiler.

In Dice-XMBD, we used a U-Net-based pixel classification model to classify individual pixels of an IMC image to their cellular origins, namely, nuclei, cytoplasm/membrane, or background. The classification model outputs pixel-level probability values for each class, which were then input to CellProfiler (version 3.1.0) to produce the final cell segmentation masks (Figure 1).

The ground truth cell segmentation of IMC images is in general not available. To obtain the training labels, we generated pixel probability maps using an iterative manual annotation process with Ilastik on the training IMC dataset. Furthermore, the same iterative manual annotation process was performed on the testing IMC datasets to produce the ground truth pixel probability maps, which were used by CellProfiler to produce the ground truth cell segmentation masks for performance evaluation.

Note that to obtain a generic pixel classifier that can be used across IMC datasets of different channel configurations, channels of different proteins were combined based on their cellular origins into two channels, namely, nuclear and cell (membrane/cytoplasmic) channels. Channels of proteins without specific cellular locations were ignored by Dice-XMBD. The pixel classification model was trained using the combined two-channel images as input. Likewise, the same preprocessing was used at the prediction stage to produce the two-channel (nuclear/cell) images as input to the pixel classification model. Of note, although the prediction may be performed on images with different markers, the channels were always combined based on their origins so that pixel classification was performed based on the two channels of putative protein locations rather than channels of individual proteins.

### 2.2. Training and Evaluation Datasets

We used four IMC image datasets in this study. BRCA1 and BRCA2 (Ali et al., 2020) contain 548 and 746 images from patients with breast cancer with 36 and 33 markers, respectively. T1D1 (Damond et al., 2019) and T1D2 (Wang et al., 2019) contain 839 and 754 images from patients with type I diabetes with 34 markers. Dice-XMBD was trained on a subset of BRCA1 dataset (*n* = 348) with 200 held-out images reserved for validation and testing. To test the generalization ability of Dice-XMBD, we also tested the trained model on the other three independent IMC datasets (BRCA2, T1D1, and T1D2).

### 2.3. Generating Ground Truth Cell Masks

The ground truth pixel probability maps and the cell masks used for model training and evaluation were generated using Ilastik and CellProfiler. We used the smallest brush size (1 pixel) in annotating the image to avoid annotating a group of neighboring pixels of different classes. To mitigate the manual workload, the annotation was performed in an interactive manner, where the random forest prediction model of Ilastik was updated regularly during annotation to produce an uncertainty map indicating the confidence level of the classification results produced by the prediction model. The annotation was then guided by the uncertainty map to focus on the regions with high uncertainty iteratively, until the overall uncertainty values were low except for regions of which the boundaries were visually indistinguishable.

The initial annotation was performed on a randomly selected subset of the dataset. After the initial annotation, we loaded all the images from the dataset into Ilastik to calculate their uncertainty maps, and then selected those with the highest average uncertainty values for further annotation. This process was iterated until the uncertainty values of all images converged, that is, the average uncertainty value over all images did not decrease significantly for three consecutive iterations.

In the end, we annotated 49 images in BRCA1 to train the model in Ilastik. We then imported all the images of the BRCA1 dataset into Ilastik for batch processing and export their corresponding pixel classification probability maps for training Dice-XMBD. The probability maps were further input to CellProfiler to produce the ground truth cell segmentation. In CellProfiler, we used the “IdentifyPrimaryObjects” module to segment the cell nuclei and used the “IdentifySecondaryObjects” to segment the cell membranes using the propagation method. The output masks from CellProfiler are regarded as ground truth cell segmentation of the dataset for performance evaluation.

We also generated the ground truth cell masks of the other three datasets by the same iterative procedure separately for testing the generalization ability of Dice-XMBD. During the process, 72 images in BRCA2, 39 images in T1D1, and 67 images in T1D2 were manually annotated.

### 2.4. Training the U-Net Cell Segmentation Model

#### 2.4.1. Image Preprocessing

The multiplexed IMC images were first merged into two channels by averaging the per-pixel values from the selected membrane and nuclear channels. After merging channels, the input IMC images were then preprocessed by hot pixel removal, dynamic range conversion, normalization, and image cropping/padding into fix-sized patches. First, we applied a 5 × 5 low-pass filter on the image to remove hot pixels. If the difference between an image pixel value and the corresponding filtered value was larger than a preset threshold (50 in our experiments), the pixel would be regarded as a hot pixel and its value would be replaced by the filtered value. As the dynamic range of pixels values differs among IMC images of different batches and different channels, we further min-max normalized all images to [0,255] to remove such batch effect as:


(1) $$\begin{array}{r} x_{ij}^{{\;}^{\prime }}=\frac{x_{ij}-X_{min}}{X_{max}-X_{min}}*255, \end{array}$$

where *x*_*ij*_ denotes the pixel value in one channel, and *X*_*max*_ and *X*_*min*_ denote the maximum and minimum values in the channel. Of note, as the pixel values in IMC images have a high dynamic range, transforming the pixel values from its dynamic range to [0, 255] would suffer from detail suppression by one or few extremely large values. Therefore, we thresholded the image pixel values at 99.7% percentile for each image before normalization.

Finally, we merged all the nuclear channels into one consolidated nuclear channel, and membrane/cytoplasmic channels into one cell channel, by averaging on all channel images with pre-selected sets of protein markers, respectively. We converted the merged two-channel images into patches of 512 × 512 pixels. Image boundary patches that are smaller than the target patch size are padded to target size. For the padded pixels, we set the pixel values of both channels to 0 and the pixel type as background.

#### 2.4.2. Data Augmentation

Data augmentation is an effective strategy to reduce overfitting and enhance the robustness of the trained models, especially when training data are insufficient. We applied the following data augmentation methods on the input images before feeding to our U-Net-based pixel classification network.

First, photometric transformations including contrast stretching and intensity adjustments were used. For contrast stretching, we changed the level of contrast by multiplication with a factor randomly drawn from the range of [0.5, 1.5]. Similarly, for intensity adjustments we changed the level of intensities by multiplication with a factor randomly drawn from the range of [0.5, 1.5]. Geometric transformations including image flipping and rotation were used. For flipping, we implemented random horizontal or vertical flipping. For rotation, the rotating angle is randomly distributed in the range of [−180, 180]. Note that geometric transformations were applied to pairs of input and output images of the network. We also injected random Gaussian noise to the two input channels of the input images. Examples of data augmentation are shown in Supplementary Figures 1, 2.

#### 2.4.3. Constructing a Pixel Classification Model

The U-Net pixel classification network is an end-to-end fully convolutional network and contains two paths. The contracting path (or the encoder) uses a typical CNN architecture. Each block in the contracting path consists of two successive 3 × 3 convolution layers followed by a Rectified Linear Unit (ReLU) activation and a 2 × 2 max-pooling layer. This block is repeated four times. In the symmetric expansive path (or the decoder), at each stage the feature map is upsampled using 2 × 2 up-convolution. To enable precise localization, the feature map from the corresponding layer in the contracting path is cropped and concatenated onto the upsampled feature map, followed by two successive 3 × 3 convolutions and ReLU activation. At the final stage, an additional 1 × 1 convolution is applied to reduce the feature map to the required number of output channels. Three output channels are used in our case for nuclei, membrane, and background, respectively. As we output the probability map, the values are converted into the range of [0, 1] using the Sigmoid function.

#### 2.4.4. Loss Function

We take the binary cross-entropy (BCE) as the loss function, which is defined as:


(2) $$\begin{array}{r} loss\left(y,ŷ\right)=-\frac{1}{N}\overset{N}{\underset{i=0}{\sum }}\left(y_{i}*log\left(\hat{y_{i}}\right)+\left(1-y_{i}\right)*log\left(1-\hat{y_{i}}\right)\right), \end{array}$$

where *N* represents the total number of pixels in an image, *y*_*i*_ denotes the ground truth pixel probability, and $\hat{y_{i}}$ denotes the predicted pixel probability. The cross-entropy loss compares the predicted probabilities with the ground truth values. The loss is minimized during the training process.

### 2.5. Model Evaluation

In a binary cell mask, “1” represents cell boundary and “0” denotes cell interior or exterior. For every pixel in an image, true positive (TP) and true negative (TN) mean that the predicted pixel classification is the same as its label in the labeled (i.e., the ground truth) mask, while false positive (FP) and false negative (FN) mean that a pixel is misclassified. To evaluate pixel-level accuracy, we calculated the number of TP pixels and FP pixels based on the predicted and labeled binary masks.

We further evaluated model performance at the cell level. We calculated the intersection over union (IOU) on cells from predicted and labeled cell masks to determine if they are the same cell, and then counted the TP and FP cells. First, we filtered out all cells with IOU below 0.1 from the predicted cells. These cells are identified as FPs. The other cells from the predicted cell mask could be either TP or FP. If a predicted cell only overlaps with one true cell (i.e., a cell from the labeled cell mask), we assume that the cell is segmented accurately (TP). If a true cell cannot find a predicted cell, the “missing” cell is denoted as FN. When multiple predicted cells are assigned to the same true cell, we consider this as a split error. If multiple true cells are matched to the same predicted cell, we consider those predicted cells as merge errors. For simplicity, split errors and merge errors are counted as FPs. Four standard indices are measured as follows:


(3) $$\begin{array}{rcl} Recall & = & \frac{TP}{TP+FN}, \end{array}$$

(4) $$\begin{array}{rcl} Precision & = & \frac{TP}{TP+FP}, \end{array}$$

(5) $$\begin{array}{rcl} F1score & = & \frac{2*Precision*Recall}{Precision+Recall}, \end{array}$$

(6) $$\begin{array}{rcl} Jaccard & = & \frac{TP}{TP+FP+FN}. \end{array}$$

To investigate the effect of different segmentation methods on downstream analysis, an unsupervised clustering method (Phenograph Levine et al., 2015, Python package, v1.5.7) was applied to the high-dimensional single cell expression data processed from each different method under comparison, and the labeled ground truth cell mask, separately. Prior to clustering, single cell protein expressions were quantified by the mean pixel values, and then these values were censored at 99th percentile and transformed with arcsinh function. Scaled high-dimensional single cells were clustered into several groups based on selected markers as from the original publication of each individual dataset. Based on the expressions of cell-specific markers, the cell types of the clusters were identified among T cells (CD3), CD4 T cell (CD4), CD8 T cell (CD8a), B cell (CD20), macrophage (CD68), endothelial cell (CD31), and so on. By comparing the cell annotation from different segmentation methods (predicted cell mask) and the labeled cell mask, the cell annotation accuracy was calculated as *n*_*same*_/*N*_*total*_. Here, *n*_*same*_ is the number of correctly predicted cells, which are cells that correctly overlapped with the corresponding cells in the labeled mask (i.e., TP cells), and annotated to the same cell types, *N*_*total*_ is the total number of cells from the predicted mask.

## 3. Results

### 3.1. Dice-XMBD Enables Automatic Cell Segmentation

We trained our U-Net cell segmentation model using the BRCA1 dataset with 348 images as the training set and 100 images as the validation set. A complete held out test set with 100 images was used to test model performance within one dataset. We further applied the trained model directly on the other three IMC image datasets to evaluate the cross-dataset performance of the model. For performance evaluation, we computed standard indices (Recall, Precision, F1-score, and Jaccard index) for both pixel-level and cell-level accuracies (see section 2).

We compared Dice-XMBD with a generic whole-cell segmentation method across six imaging platforms, Mesmer (Greenwald et al., 2021), which used a deep learning-based algorithm trained on a large, annotated image dataset to segment single cells and nuclei separately. A trained Mesmer model was tested with combined nuclear and cell channels, which is the same as the input to Dice-XMBD. Meanwhile, we compared with three commonly used segmentation methods implemented in CellProfiler with default parameters: distance, watershed, and propagation. These methods first locate nuclei as primary objects, and then the membrane proteins are added together into an image as input to recognize cells. The distance method does not use any membrane proteins information and simply defines cell membrane by expanding several pixels around nuclei. The watershed method computes intensity gradients on the Sobel transformed image to identify boundaries between cells (Vincent and Soille, 1991), while the propagation method defines cell boundaries by combining the distance of the nearest primary object and the intensity gradients of cell membrane image (Jones et al., 2005). Hereafter, we refer to these three CellProfile-based methods as CP_distance, CP_watershed, and CP_propagation, respectively.

**Figure 2:**
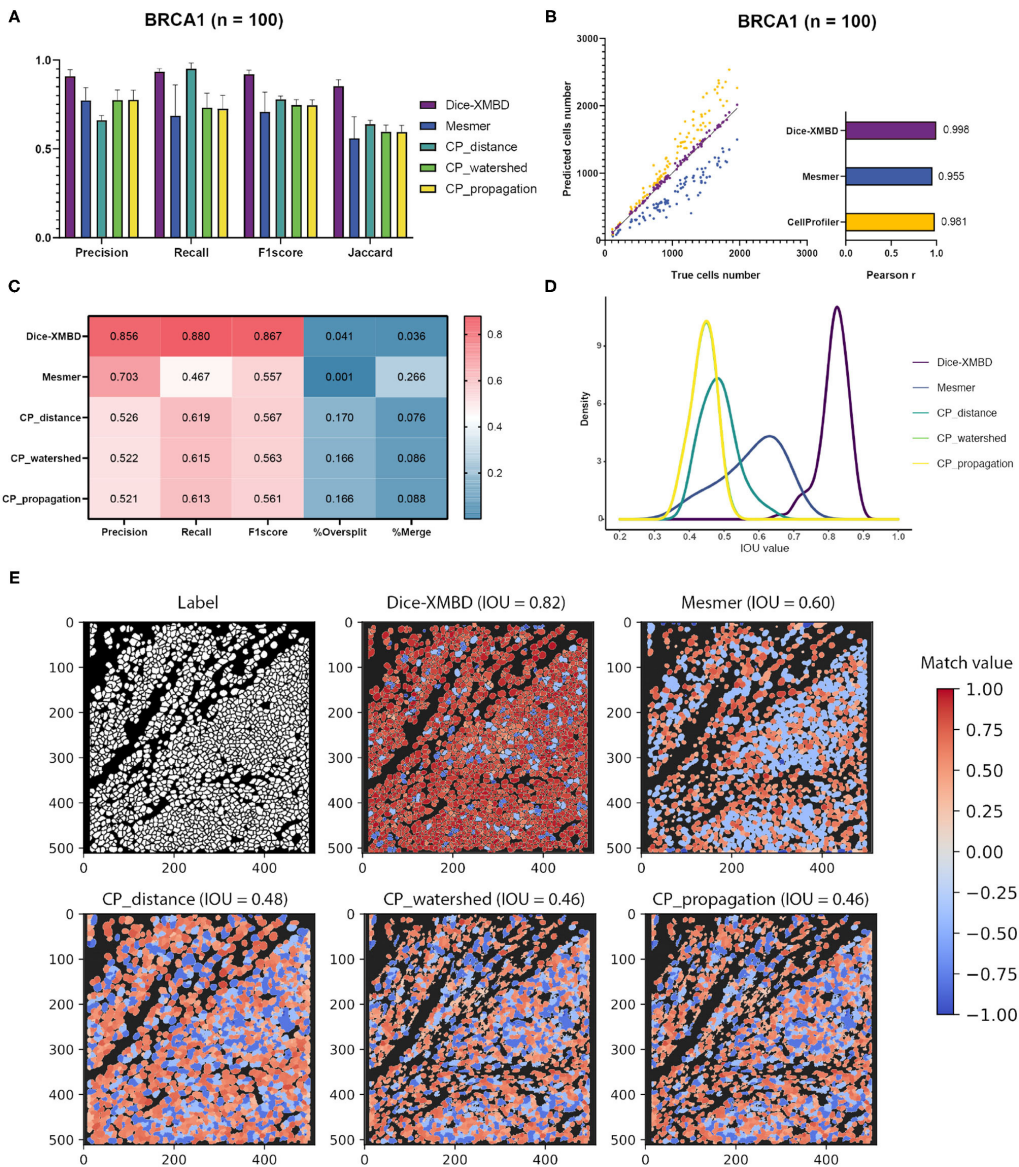
Dice-XMBD enables automatic single cell segmentation. **(A)** Pixel prediction performance comparison of Dice-XMBD, Mesmer, and CellProfiler (CP_distance, CP_watershed, CP_propagation). All data in bar plots are presented as mean ±SD. **(B)** Pearson correlations between the number of predicted cells and labeled cells per image. Note that the number of cells predicted from three CellProfiler methods are the same (here denoted as CellProfiler). **(C)** Cell prediction performance comparison. %Oversplit and %Merge denote the percentage of oversplits and merge errors in predictions. **(D)** Density plots showing the distribution of mean IOU values of matched cells per image. Note that the plots for CP_watershed and CP_propagation overlapped. **(E)** An example of labeled and predicted single cell masks from benchmarked methods. The title of each subfigure shows the method and the mean IOU value of all matched cell pairs in the predicted mask with regard to the labeled cell mask. Match value represents the IOU value for one-to-one cell pairs identified in the labeled and predicted cell masks. Note that computed IOU values are in the range of [0,1]. To better visualize FP cells, we use –0.4 and –0.8 to represent merged cells (multiple true cells matched to one predicted cell) and split cells (multiple predicted cells matched to one true cell), and –1 to represent all other FP cells in the predicted mask.

Results show that Dice-XMBD outperformed all other benchmarked methods with highest accuracy on pixel level (F1 score = 0.92, Jaccard index = 0.85) (Figure 2A). We also observed that CP_distance obtained the highest recall (Recall = 0.95) but lowest precision (Precision = 0.66), which means that it can identify almost every pixel correctly in the labeled mask but only 66% of predicted pixels were accurate.

In terms of cell-level performance, we first counted cells per image from predicted and labeled cell masks. The prediction result from Dice-XMBD showed highest correlation with the ground truth (Pearson correlation = 0.998) among all methods tested. Mesmer (Pearson correlation = 0.955) and CellProfiler (Pearson correlation = 0.981) also achieved high correlation with the ground truth. However, Mesmer tended to predict less cells while CellProfiler was more likely to over-split cells, as shown in Figures 2B,C. Moreover, Figure 2C shows that Dice-XMBD had the best prediction performance (F1-score = 0.867) considering precision (Precision = 0.856, percent of cells that were correctly predicted) and recall (Recall = 0.880, percent of true cells that are predicted) than Mesmer (F1-score = 0.557) and CellProfiler (F1-score = 0.567, 0.563, and 0.561 for CP_distance, CP_watershed, and CP_propagation, respectively). We further checked the IOU distribution of all one-to-one cell pairs (predicted and true cells), Figure 2D demonstrates that most matched cell pairs predicted from Dice-XMBD were highly overlapping (mean = 0.815, median = 0.821), followed by Mesmer where most matched pairs are only half area of overlap (mean = 0.579, median = 0.595). An example of BRCA1 shown in Figure 2E demonstrates that Dice-XMBD prediction was far superior to other benchmarked methods since it contained most cells with high matched values.

### 3.2. Dice-XMBD Enables Generic IMC Image Segmentation

**Figure 3:**
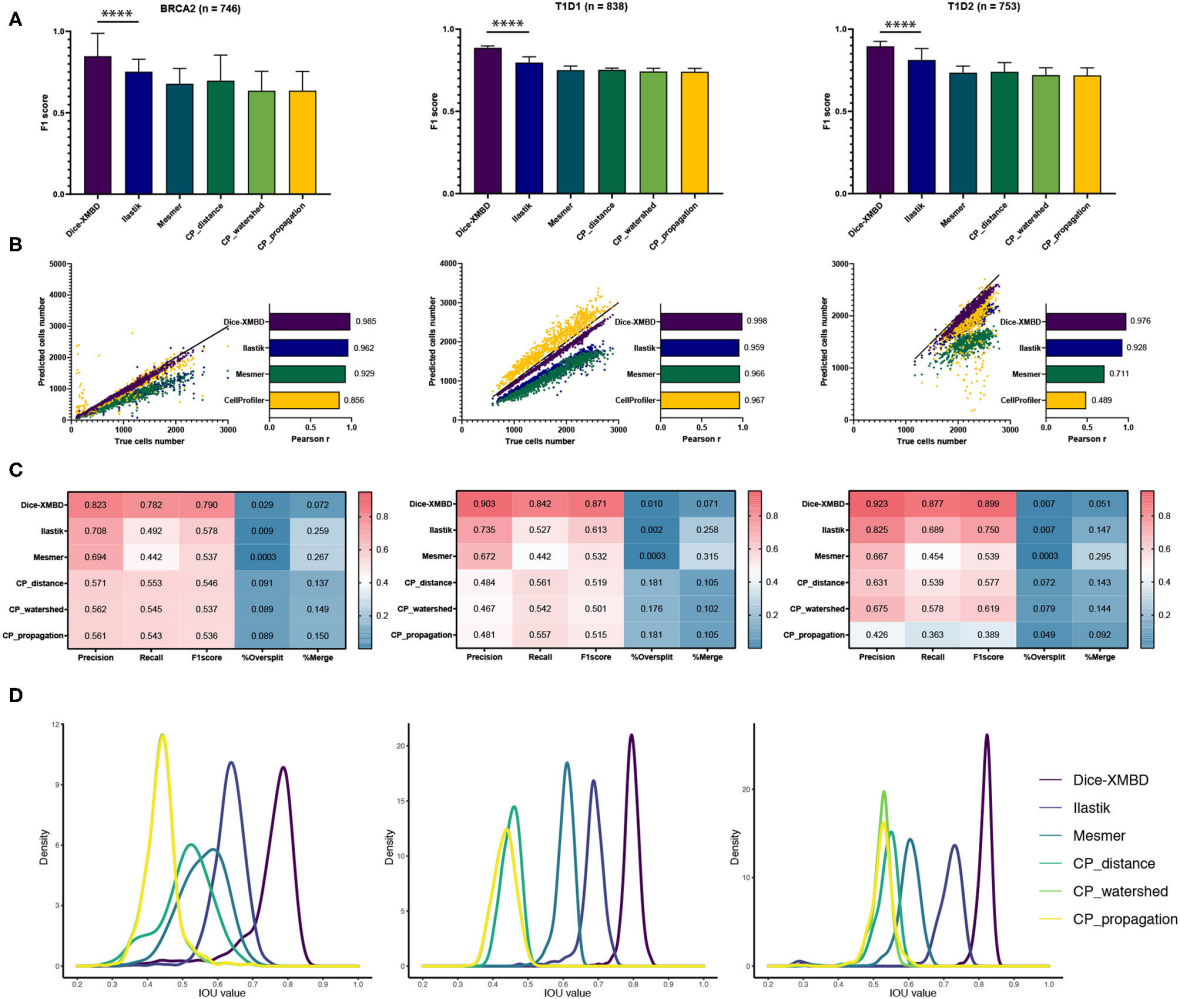
Dice-XMBD enables generic imaging mass cytometry (IMC) image segmentation. Left: BCRA2, middle: T1D1, right: T1D2. **(A)** Pixel prediction performance comparison of Dice-XMBD, Ilastik, Mesmer, and CellProfiler (CP_distance, CP_watershed, CP_propagation). All data in bar plots are presented as mean ±SD. **(B)** Pearson correlations between the number of predicted cells and labeled cells per image. Note that the number of cells predicted from three CellProfiler methods are the same (here denoted as CellProfiler). **(C)** Heatmaps of cells prediction performance of six benchmarked methods. %Oversplit and %Merge denote the percentage of oversplits and merge errors in predictions. **(D)** Density plots showing the distribution of mean IOU values of matched cells per image. Note that the plots for CP_watershed and CP_propagation overlapped for BRCA2 and T1D1.

The key idea of this study was to generate an IMC-specific single cell segmentation model across different datasets with multiple proteins. We selected three independent IMC datasets generated from different labs to test the generalization ability of Dice-XMBD. Apart from the benchmarked methods mentioned above, we also included the Ilastik model trained from BRCA1 annotations in our comparison. Figure 3A shows that Dice-XMBD outperformed all the other methods, followed by Ilastik. Moreover, the performance of cells prediction from Dice-XMBD was the best and the most stable for all three datasets, while Ilastik and Mesmer tended to under-predict cells. CellProfiler predicted less cells in BRCA2 and over-predicted cells in two T1D datasets, as shown in Figures 3B,C. Furthermore, Dice-XMBD predictions contained most of the cells with IOU value higher than 0.8 (Figure 3D and Supplementary Figure 3).

### 3.3. Dice-XMBD Enables Accurate Downstream Biological Analysis

**Figure 4:**
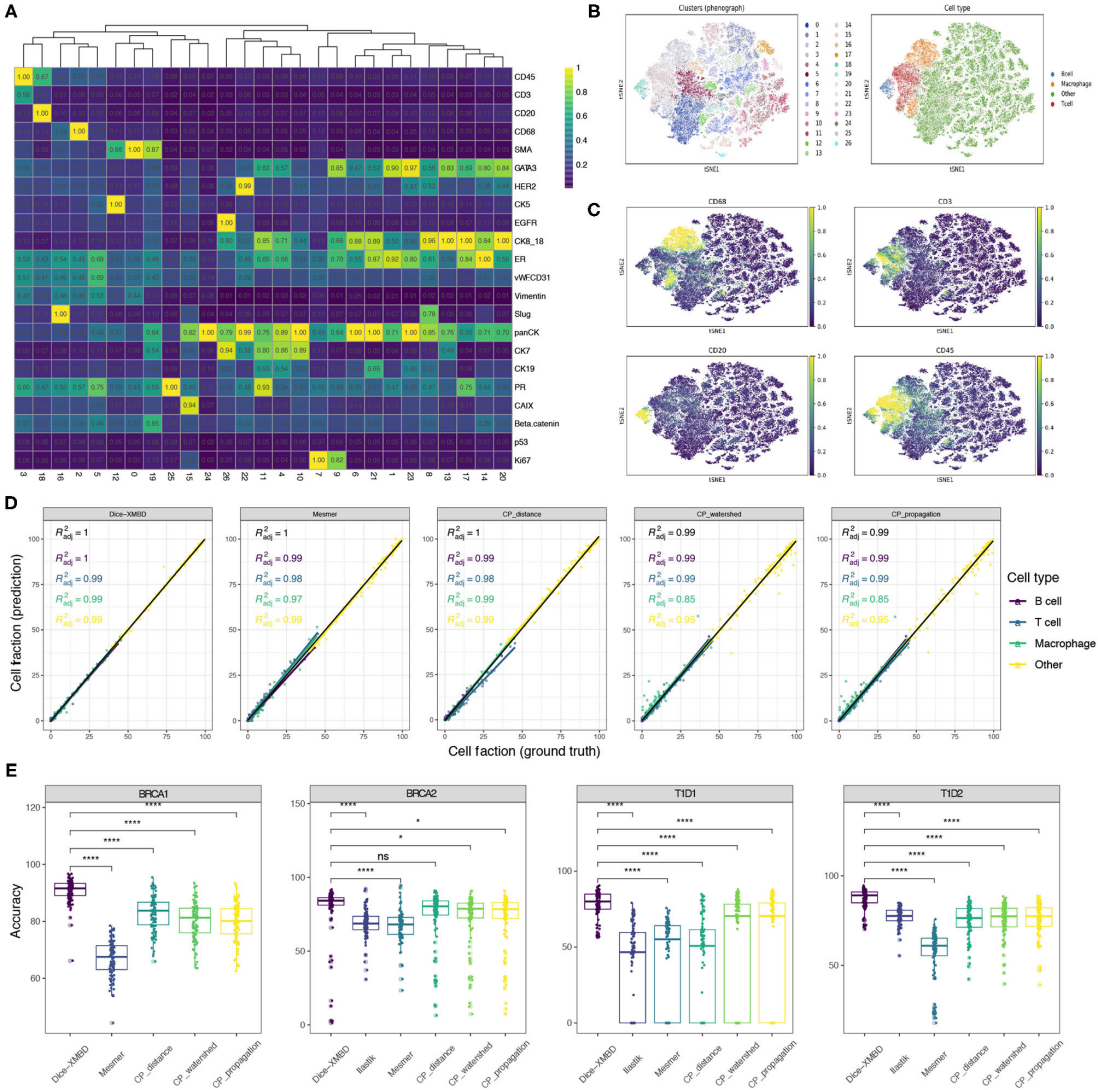
Dice-XMBD enables accurate downstream biological analysis. **(A)** Heatmap showing median values of normalized markers expression in each Phenograph cluster. **(B)** tSNE map representing high-dimensional single cells colored by Phenograph clusters (left) and cell types (right). **(C)** tSNE map representing single cells colored by cell-type-specific markers expression (CD68 for macrophage, CD45 and CD3 for T cells, CD45 and CD20 for B cells). Single cells on **(A–C)** were from BRCA1 dataset and segmented by Dice-XMBD. **(D)** Scatter plots of cell fraction obtained from ground truth (*x*-axis) and five segmentation methods (*y*-axis), colored by different cell types identified from BRCA1 dataset. **(E)** Cell annotation accuracy from Dice-XMBD and other benchmarked methods in four datasets. Pairwise comparisons of Dice-XMBD and other methods: *P < 0.05; ****P < 0.0001; n.s., not significant (Student's *t*-test).

To investigate the influence of segmentation accuracy on downstream analysis, we clustered single cells resulting from different segmentation methods separately using Phenograph and compared the clustering results. Taking the result from single cells obtained from Dice-XMBD segmentation on BRCA1 dataset as an example, these cells can be clustered into 26 distinct clusters [Figure 4A, t-distributed stochastic neighbor embedding (t-SNE) visualization in Figure 4B]. Based on the scaled mean expression for each cluster, we were able to annotate Cluster 3 as T cells, Cluster 18 as B cells, Cluster 16 as macrophage, and the remaining clusters to other cell types which may include tumor cells, stromal cells, or endothelial cells (Figure 4C). We performed the same clustering and annotation process on single cells obtained from other segmentation methods and the ground truth segmentation on all three datasets separately as well. For two T1D datasets [T1D1 (Damond et al., 2019) and T1D2 (Wang et al., 2019)], CD4 T cells, CD8 T cells, and CD31+ endothelial cells were identified based on their selected markers.

We compared the concordance of cell fractions based on annotations from different segmentation methods (prediction) versus those from ground truth segmentation (ground truth) (Figure 4D and Supplementary Figures 4A–7A). On BRCA1 dataset, Dice-XMBD performed better compared with all other segmentation methods on overall results and results of certain cell types (Figure 4D). Significantly, two CellProfiler-based methods (CP_watershed, *R*^2^ = 0.85 and CP_propagation, *R*^2^ = 0.85) showed inferior performance in reproducing cell fraction results in macrophage while Dice-XMBD still achieved an *R*^2^ = 0.99 in this cell types. CP_distance delivered reasonable performance in macrophage, but was still inferior to Dice-XMBD on T cell. Similar results can be observed on other datasets as well. For example, for the T1D1 dataset, CD4 T cells were poorly predicted by Ilastik (*R*^2^ = 0.043) and CP_distance (*R*^2^ = 0.055) (Supplementary Figure 6A). For the T1D2 dataset, endothelial cells were poorly predicted by Ilastik (*R*^2^ = 0.58) and macrophage cells were poorly predicted by Mesmer (*R*^2^ = 0.033). On the other hand, Dice-XMBD delivered highly consistent prediction results across all cell types in all datasets except for T cell in BRCA2 dataset, where all methods did not perform well.

In addition to cell fraction, we also evaluated the annotation accuracy of individual cells for each method (Figure 4E and Supplementary Figures 4B–7B), which is important for spatially related analysis of single cell data such as neighborhood analysis. Dice-XMBD achieved the highest cell annotation accuracies among all segmentation methods on overall results (Figure 4E), and performed as well as or better than other methods on all individual cell types in all datasets (Supplementary Figures 4B–7B).

### 3.4. Generalization Ability of Dice-XMBD

To investigate the impact of the training data on the segmentation performance of Dice-XMBD, we trained Dice-XMBD using different training datasets, and evaluated the performance of the resulting models on other IMC datasets used in this study. Results show that segmentation performance in terms of pixel-level accuracy were in fact very similar among these models (Supplementary Tables 1–4). We further asked if the performance of Dice-XMBD could be improved by training on multiple datasets. Interestingly, the model did not consistently perform better when more than one datasets were combined as the training set (Supplementary Tables 1–4). All together, these results suggest that by using location specific channels, Dice-XMBD were highly robust to different training datasets, and a Dice-XMBD model trained on one dataset can be well generalized to segmentation tasks on other IMC datasets.

Of note, in our approach, the channels of same locations were simply averaged without applying any weighting scheme to produce the location specific channels. We tried to min-max-normalize the selected channels before averaging so that all selected channels contributed equally to the combined channels. However, the pixel-level accuracy dropped on all datasets, albeit at different levels of degradation on different datasets (Supplementary Tables 1–4). As different channels may contain different levels of information to the final segmentation results, combining them with equal weights may not be the optimal approach. However, how to find the optimal weighting combination of different channels remains an open question that deserves further exploration.

## 4. Discussion

Highly multiplexed single cell imaging technologies such as IMC are becoming increasingly important tools for both basic biomedical and clinical research. These tools can unveil complex single-cell phenotypes and their spatial context at unprecedented details, providing a solid base for further exploration in cancer, diabetes, and other complex diseases. Nevertheless, cell segmentation has become a major bottleneck in analyzing multiplexed images. Conventional approaches rely on intensities of protein markers to identify different cellular structures such as nuclei, cytoplasm, and membrane. Unfortunately, the intensity values of these markers are strongly cell type-specific and may vary from cells to cells. In addition, the staining also shows variability across images or datasets. As a result, the accuracy and robustness of the segmentation results are far from optimal. On the other hand, high-order visual features including spatial distribution of markers, textures, and gradients are relevant to visually identify subcellular structures by human. However, these features are not considered in conventional methods to improve the cell segmentation results.

The DNN-based image segmentation approaches provide an opportunity to leverage high-order visual features at cellular level for better segmentation results. Unfortunately, they require a significant amount of annotation data that are in general difficult to acquire. In addition, the highly variable channel configurations of multiplexed images impose another important obstacle to the usability of these methods as most of them lack the ability to adapt to different channel configurations after models are trained. In this study, we develop Dice-XMBD, a generic solution for IMC image segmentation based on U-Net. Dice-XMBD overcomes the limitation of training data scarcity and achieves human-level accuracy by distilling expert knowledge from Ilastik with manual input of human as a teacher model. Moreover, by consolidating multiple channels of different proteins into two cellular structure-aware channels, Dice-XMBD provides an effective off-the-shelf solution for cell segmentation tasks across different studies without retraining that can lead to significant delay in analysis. Importantly, our evaluation results further demonstrate Dice-XMBD's good generalization ability to predict single cells for different IMC image datasets with minimum impact to downstream analysis, suggesting its values as an generic tool for hassle-free large-scale IMC data analysis. Finally, to facilitate the analysis of large amount of IMC data currently being generated around the world, we made Dice-XMBD publicly available as an open-source software on GitHub (https://github.com/xmuyulab/Dice-XMBD).

## Data Availability Statement

All datasets used for this study can be found at GitHub (https://github.com/xmuyulab/Dice-XMBD). These datasets are downloaded from: BRCA1 (https://idr.openmicroscopy.org/search/?query=Name:idr0076ali-metabric/experimentA), BRCA2 (https://zenodo.org/record/3518284#.YLnmlS8RquU), T1D1 (https://data.mendeley.com/datasets/cydmwsfztj/1), T1D2 (part1: https://data.mendeley.com/datasets/9b262xmtm9/1, part2: https://data.mendeley.com/datasets/xbxnfg2zfs/1), respectively.

## Author Contributions

WY, LW, RY, and JH discussed the ideas and supervised the study. YQ and YJ implemented and conducted experiments in deep network cell segmentation. XX performed the model evaluation and biological analysis on segmentation results. XX, WY, and RY wrote the manuscript. All authors discussed and commented on the manuscript.

## Funding

This study was funded by National Natural Science Foundation of China (grant no. 81788101 to JH).

## Conflict of Interest

RY and WY are shareholders of Aginome Scientific. The remaining authors declare that the research was conducted in the absence of any commercial or financial relationships that could be construed as a potential conflict of interest.

## Publisher's Note

All claims expressed in this article are solely those of the authors and do not necessarily represent those of their affiliated organizations, or those of the publisher, the editors and the reviewers. Any product that may be evaluated in this article, or claim that may be made by its manufacturer, is not guaranteed or endorsed by the publisher.
