# Supplementary material for: Dice-XMBD: Deep Learning-Based Cell Segmentation for Imaging Mass Cytometry
Source: Front Genet. 2021 Sep 15;12:721229. doi: 10.3389/fgene.2021.721229 (PMC8480472; doi:10.3389/fgene.2021.721229)
Supplement: Supplementary file 1 [file Data_Sheet_1.pdf]

Supplementary Material for Dice-XMBD: Deep learning-based cell segmentation for imaging mass cytometry

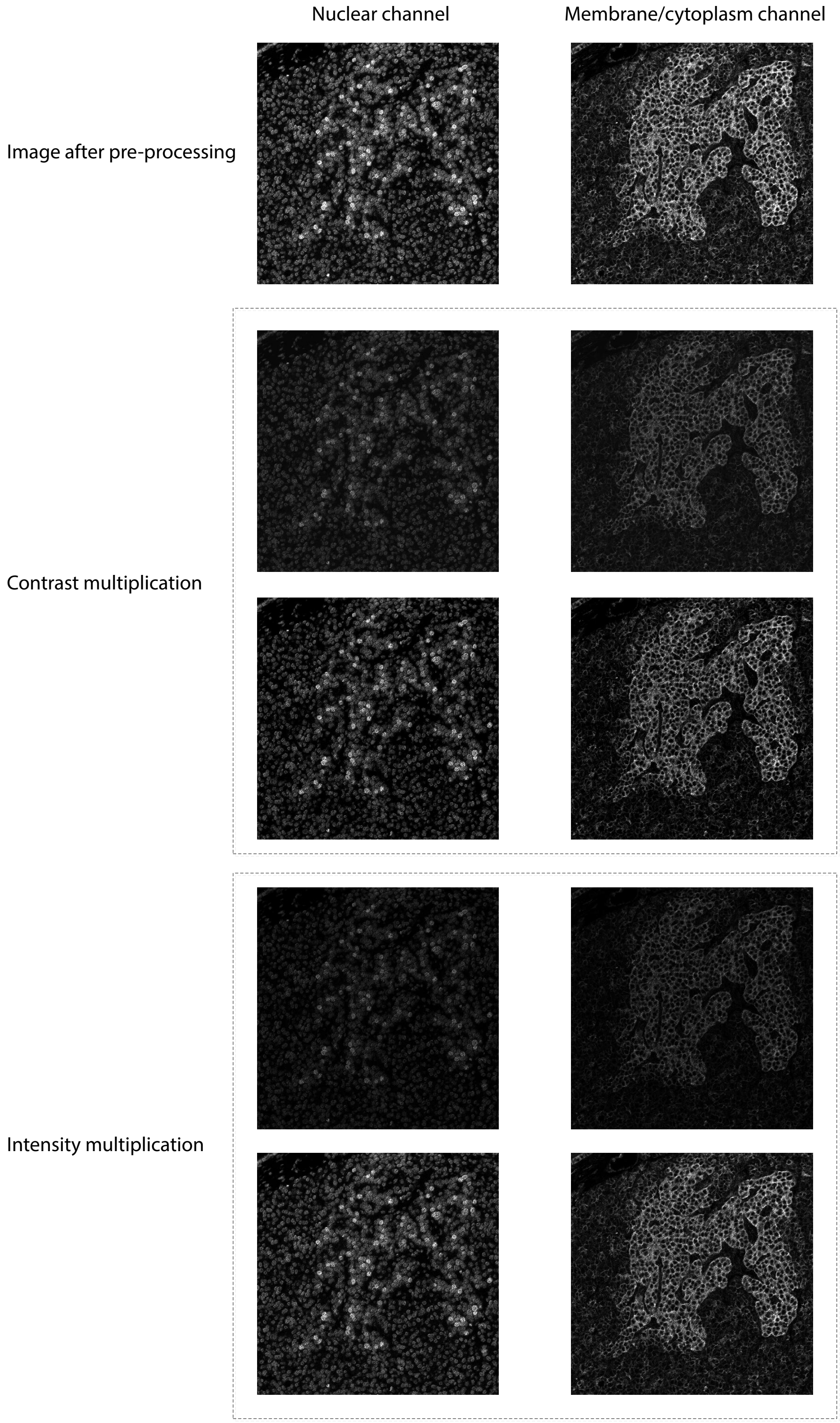

Figure S1. An example of data augmentation from T1D1 dataset: 2-channel images after pre-processing, contrast and intensity multiplication.

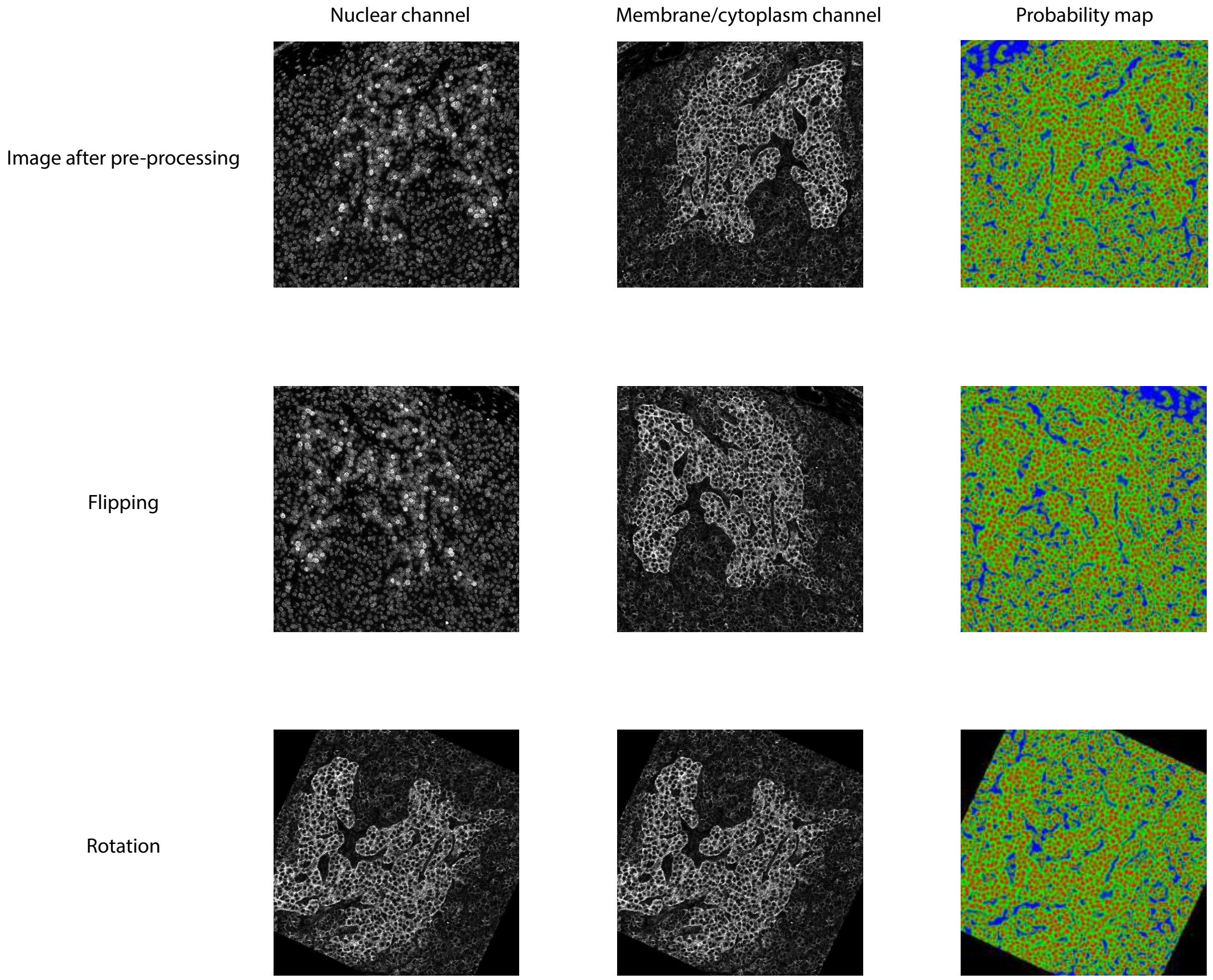

Figure S2. An example of data augmentation from T1D1 dataset: 2-channel images and probability maps after preprocessing, flipping, and rotation.

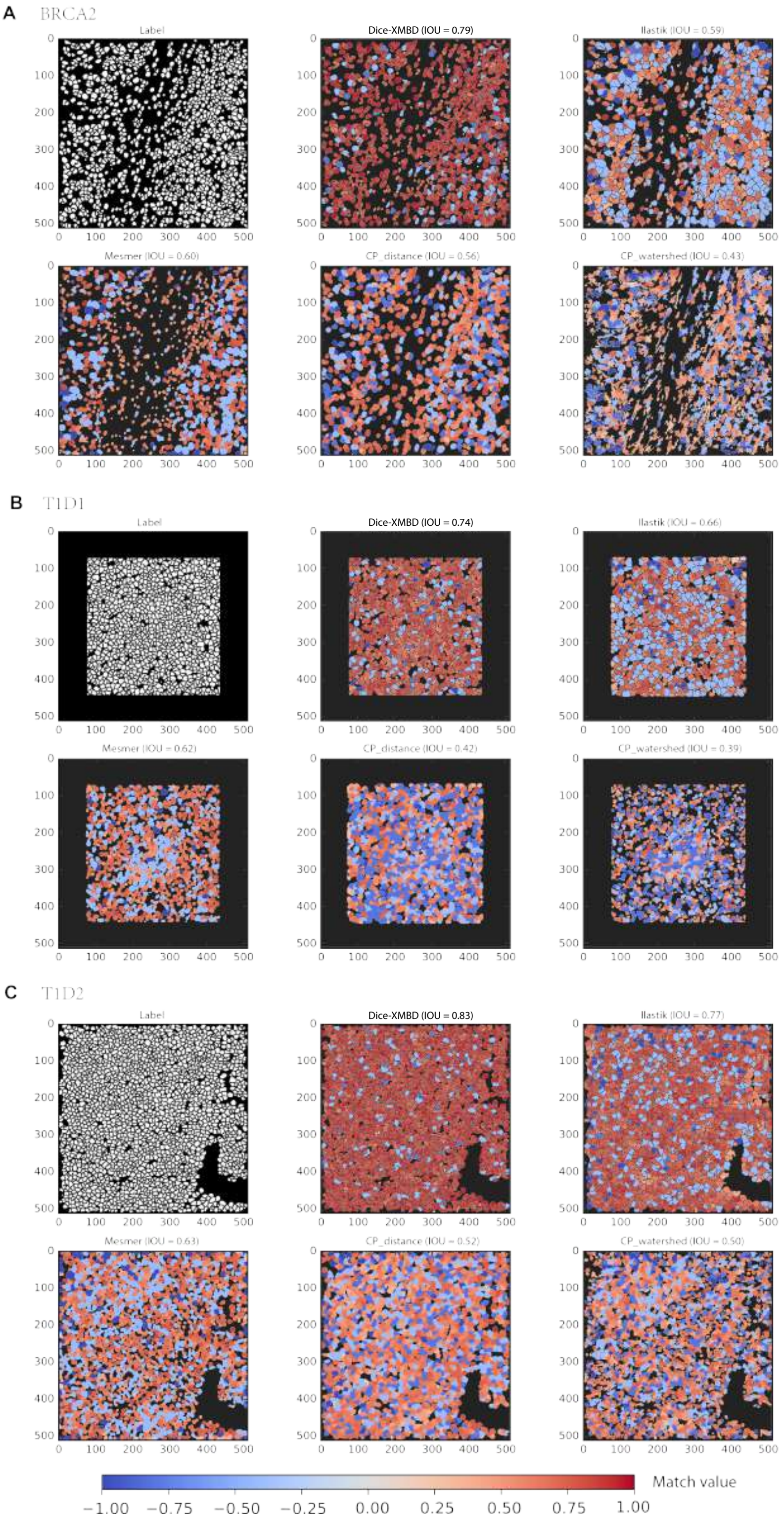

Figure S3. An example of labeled and predicted single cell masks from benchmarked methods for BRCA2 (**A**), T1D12 (**B**), and T1D2 (**C**). The title of each subfigure shows the method and the mean IOU value of all matched cell pairs in the predicted mask with regards to the labeled cell mask. Match value represents the IOU value for one-to-one cell pairs identified in the labeled and predicted cell masks. Note that computed IOU values are in the range of [0,1]. To better visualize FP cells, we use -0.4 and -0.8 to represent merged cells (multiple true cells matched to one predicted cell) and split cells (multiple predicted cells matched to one true cell), and -1 to represent all other FP cells in the predicted mask.

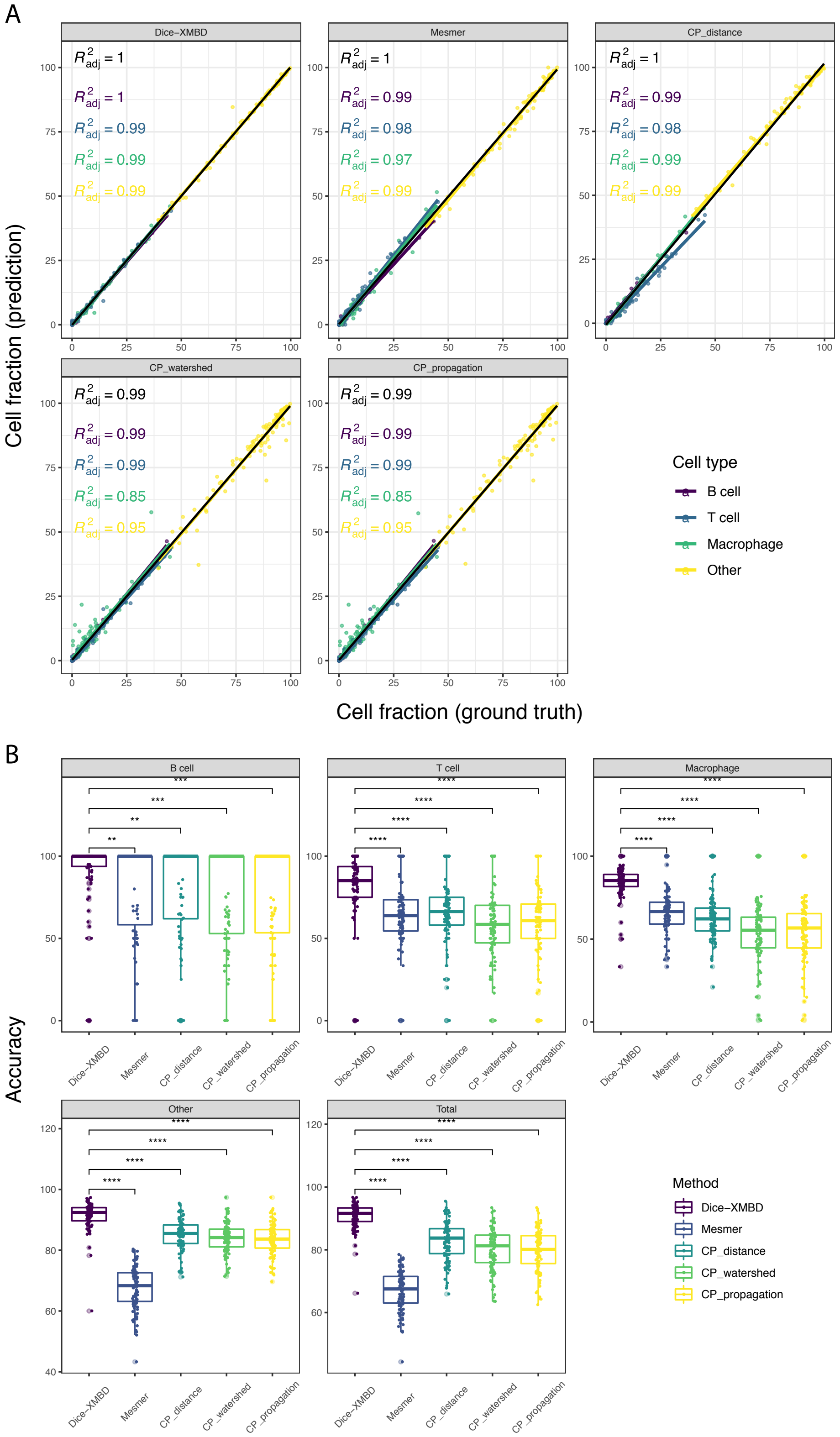

Figure S4. Cell clustering and identification performance on BRCA1. **(A)** Scatter plots of cell fraction obtained from ground truth (x-axis) and five segmentation methods (y-axis), coloured by different cell types. **(B)** Cell annotation accuracy from Dice-XMBD and other benchmarked methods. Pairwise comparisons of Dice-XMBD and other methods:*$P < 0.05$; **$P < 0.01$; ***$P < 0.001$; ****$P < 0.0001$; n.s., not significant (Student's t-test).

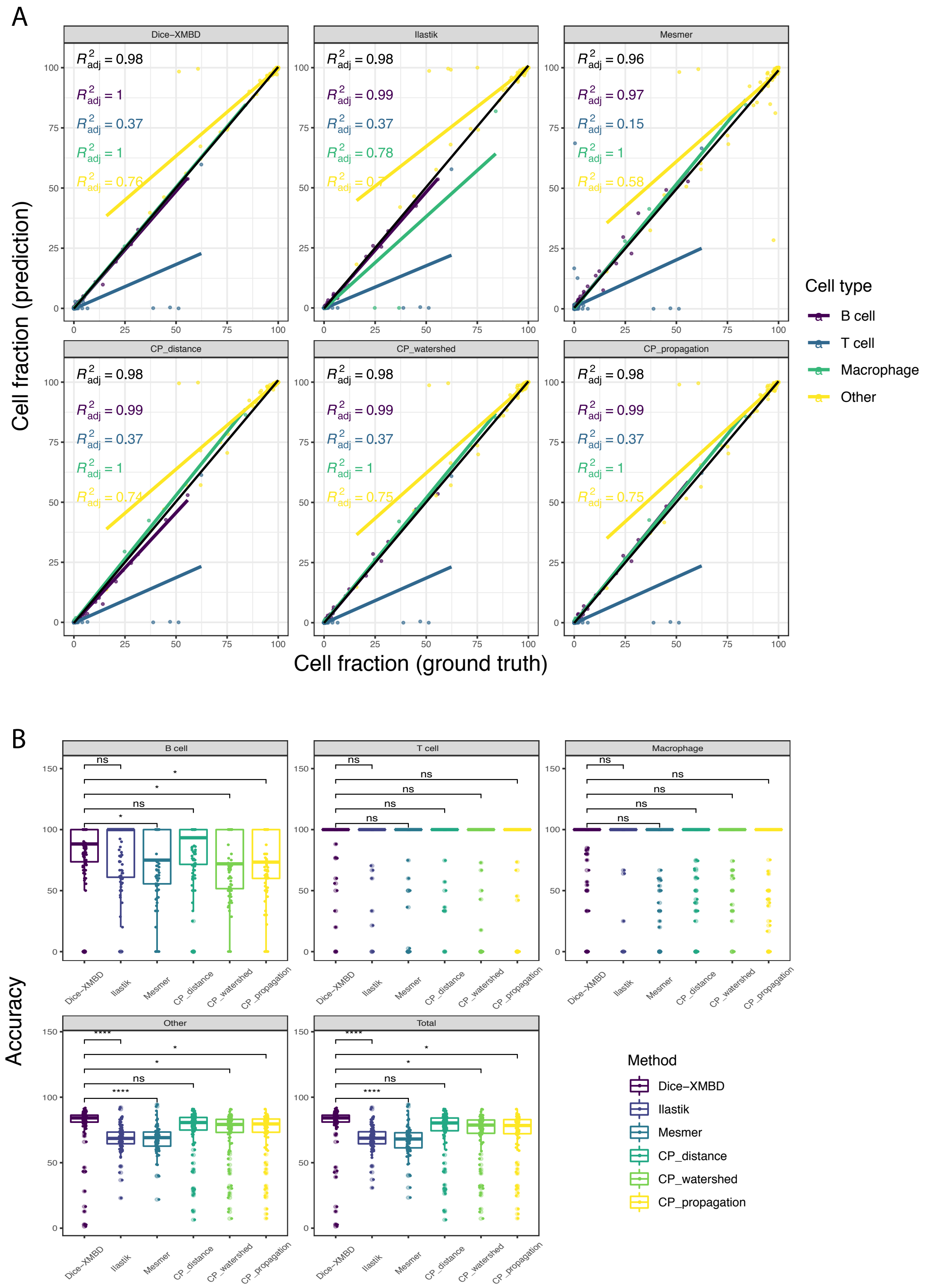

Figure S5. Cell clustering and identification performance on BRCA2. **(A)** Scatter plots of cell fraction obtained from ground truth (x-axis) and five segmentation methods (y-axis), coloured by different cell types. **(B)** Cell annotation accuracy from Dice-XMBD and other benchmarked methods. Pairwise comparisons of Dice-XMBD and other methods:*$P < 0.05$; **$P < 0.01$; ***$P < 0.001$; ****$P < 0.0001$; n.s., not significant (Student's t-test). They are less number of T cells and macrophage in both prediction and label, for this case, the accuracy of cell type in the image was set to 100.

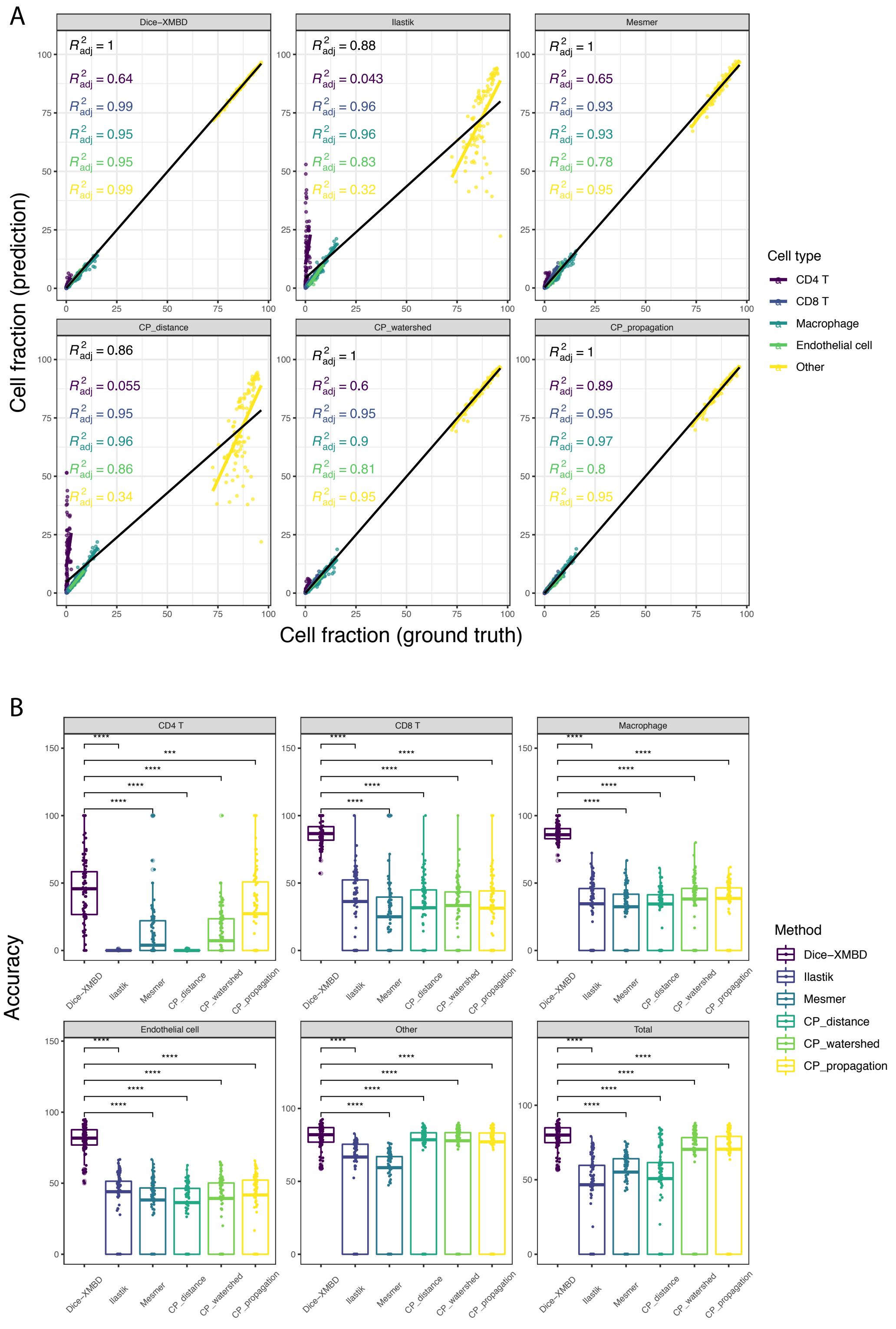

Figure S6. Cell clustering and identification performance on T1D1. **(A)** Scatter plots of cell fraction obtained from ground truth (x-axis) and five segmentation methods (y-axis), coloured by different cell types. **(B)** Cell annotation accuracy from Dice-XMBD and other benchmarked methods. Pairwise comparisons of Dice-XMBD and other methods:*$P < 0.05$; **$P < 0.01$; ***$P < 0.001$; ****$P < 0.0001$; n.s., not significant (Student's t-test).

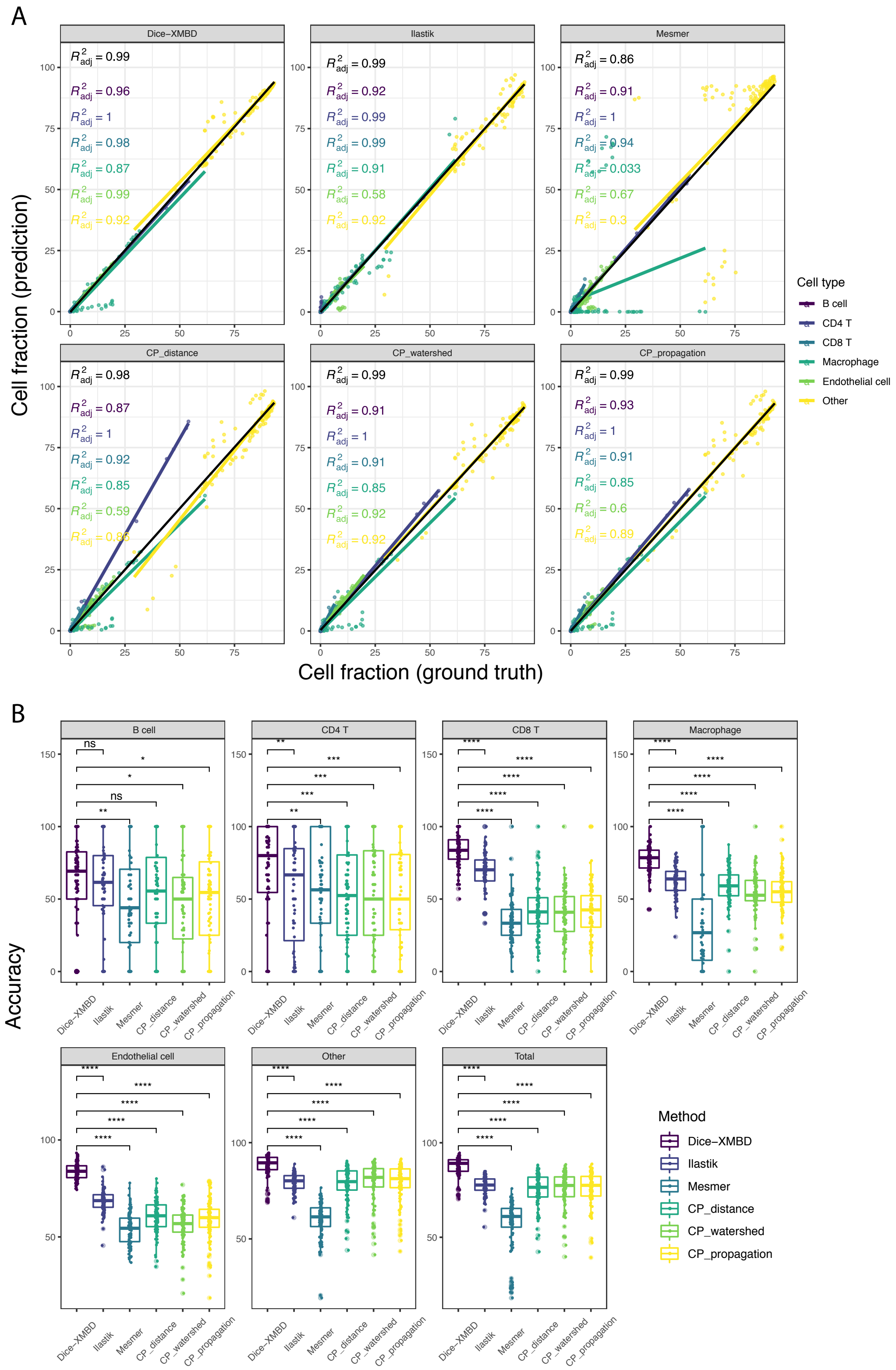

Figure S7. Cell clustering and identification performance on T1D2. **(A)** Scatter plots of cell fraction obtained from ground truth (x-axis) and five segmentation methods (y-axis), coloured by different cell types. **(B)** Cell annotation accuracy from Dice-XMBD and other benchmarked methods. Pairwise comparisons of Dice-XMBD and other methods:*$P < 0.05$; **$P < 0.01$; ***$P < 0.001$; ****$P < 0.0001$; n.s., not significant (Student's t-test).

Table S1. Pixel-level precision on 4 datasets (column) using models (row) trained with different datasets. BRCA1/BRCA2/T1D1/T1D2: models trained with each dataset separately; BRCA1/BRCA2/T1D1/T1D2 (normalized): models trained with each dataset by normalising selected channels before merging; BRCAs: models trained with two BRCA datasets; T1Ds: models trained with two T1D datasets; XXXX+XXXX+XXXX: model trained with all the datasets listed in the name (e.g., model BRCA2+T1D1+T1D2 is trained using BRCA2, T1D1, and T1D2). All data in the table are presented as mean values +/- SD.

	BRCA1	BRCA2	T1D1	T1D2	Mean
BRCA1		0.8925±0.1193	0.8770±0.0110	0.8916±0.0340	0.88702
BRCA1 (normalized)		0.8788±0.0358	0.8443±0.0115	0.8097±0.0265	0.84427
BRCA2	0.8744±0.0745		0.8759±0.0135	0.8917±0.0335	0.88065
BRCA2 (normalized)	0.6941±0.1677		0.8469±0.0138	0.7724±0.0361	0.77115
T1D1	0.8123±0.1644	0.8107±0.2287		0.9030±0.0308	0.84201
T1D1 (normalized)	0.6824±0.1975	0.7875±0.1983		0.8100±0.0304	0.75996
T1D2	0.8818±0.0726	0.8639±0.1897	0.8778±0.0128		0.87450
T1D2 (normalized)	0.7964±0.1001	0.8654±0.0890	0.8510±0.0151		0.83762
BRCAs			0.8787±0.0113	0.8904±0.0338	0.88457
T1Ds	0.8671±0.1037	0.8472±0.2051			0.85713
BRCA2+T1D1+T1D2	0.8833±0.0668				0.88334
BRCA1+T1D1+T1D2		0.8669±0.1835			0.86691
BRCA1+BRCA2+T1D2			0.8756±0.0119		0.87557
BRCA1+BRCA2+T1D1				0.8913±0.0329	0.89132

Table S2. Pixel-level recall on 4 datasets (column) using models (row) trained with different datasets. See Table S1 caption for model definitions. All data in the table are presented as mean values +/- SD.

	BRCA1	BRCA2	T1D1	T1D2	Mean
BRCA1		0.8589±0.0581	0.9124±0.0164	0.9199±0.0344	0.89705
BRCA1 (normalized)		0.8334±0.0753	0.8748±0.0156	0.8443±0.0310	0.85083
BRCA2	0.9016±0.0207		0.9089±0.0126	0.9008±0.0297	0.90379
BRCA2 (normalized)	0.8444±0.0248		0.8776±0.0143	0.8498±0.0376	0.85724
T1D1	0.8743±0.0211	0.8750±0.0480		0.8888±0.0301	0.87937
T1D1 (normalized)	0.8166±0.0209	0.8441±0.0397		0.8169±0.0314	0.82587
T1D2	0.8838±0.0218	0.8780±0.0445	0.9093±0.0166		0.89035
T1D2 (normalized)	0.8246±0.0216	0.8118±0.0536	0.8592±0.0159		0.83185
BRCAs			0.9087±0.0138	0.9079±0.0301	0.90829
T1Ds	0.8770±0.0202	0.8856±0.0427			0.88132
BRCA2+T1D1+T1D2	0.8990±0.0210				0.89904
BRCA1+T1D1+T1D2		0.8730±0.0424			0.87298
BRCA1+BRCA2+T1D2			0.9089±0.0135		0.90893
BRCA1+BRCA2+T1D1				0.9043±0.0302	0.90429

Table S3. Pixel-level F1 score on 4 datasets (column) using models (row) trained with different datasets. See Table S1 caption for model definitions. All data in the table are presented as mean values +/- SD.

	BRCA1	BRCA2	T1D1	T1D2	Mean
BRCA1		0.8789±0.1005	0.9042±0.0105	0.9152±0.0307	0.89944
BRCA1 (normalized)		0.8535±0.0551	0.8592±0.0098	0.8264±0.0253	0.84635
BRCA2	0.8856±0.0587		0.8920±0.0098	0.8960±0.0294	0.89120
BRCA2 (normalized)	0.7489±0.1349		0.8619±0.0100	0.8084±0.0293	0.80639
T1D1	0.8288±0.1325	0.8169±0.2094		0.8958±0.0289	0.84718
T1D1 (normalized)	0.7241±0.1579	0.7973±0.1616		0.8131±0.0264	0.77819
T1D2	0.8805±0.0557	0.8530±0.1716	0.8932±0.0121		0.87559
T1D2 (normalized)	0.8063±0.0637	0.8340±0.0664	0.8549±0.0115		0.83174
BRCAs			0.8934±0.0101	0.8989±0.0298	0.89614
T1Ds	0.8670±0.0846	0.8453±0.1885			0.85615
BRCA2+T1D1+T1D2	0.8893±0.0521				0.88931
BRCA1+T1D1+T1D2		0.8547±0.1655			0.85471
BRCA1+BRCA2+T1D2			0.8919±0.0101		0.89187
BRCA1+BRCA2+T1D1				0.8976±0.0299	0.89762

Table S4. Pixel-level jaccard index on 4 datasets (column) using models (row) trained with different datasets. See Table S1 caption for model definitions. All data in the table are presented as mean values +/- SD.

	BRCA1	BRCA2	T1D1	T1D2	Mean
BRCA1		0.7824±0.1115	0.8127±0.0167	0.8313±0.0394	0.80880
BRCA1 (normalized)		0.7477±0.0704	0.7533±0.0149	0.7048±0.0310	0.73527
BRCA2	0.7981±0.0676		0.8052±0.0157	0.8127±0.0373	0.80532
BRCA2 (normalized)	0.6140±0.1439		0.7574±0.0152	0.6794±0.0368	0.68358
T1D1	0.7235±0.1410	0.7264±0.2038		0.8122±0.0361	0.75402
T1D1 (normalized)	0.5874±0.1612	0.6854±0.1684		0.6858±0.0324	0.65286
T1D2	0.7897±0.0650	0.7690±0.1700	0.8072±0.0195		0.78864
T1D2 (normalized)	0.6796±0.0785	0.7198±0.0801	0.7468±0.0174		0.7154
BRCAs			0.8075±0.0163	0.8173±0.0382	0.81242
T1Ds	0.7718±0.0901	0.7621±0.1851			0.76697
BRCA2+T1D1+T1D2	0.8035±0.0610				0.80349
BRCA1+T1D1+T1D2		0.7698±0.1635			0.76976
BRCA1+BRCA2+T1D2			0.8050±0.0162		0.80500
BRCA1+BRCA2+T1D1				0.8153±0.0379	0.81528
